# ASSOCIATION BETWEEN COGNITIVE DYSFUNCTION AND DIABETES IN PATIENTS OVER 65 YEARS OLD: A CROSS-SECTIONAL STUDY USING PROPENSITY SCORE MATCHING

**DOI:** 10.2340/jrm.v56.18372

**Published:** 2024-02-21

**Authors:** Liwen ZHAI, Yao YANG, Jun ZHANG, Weiqian HOU, Yujie YANG, Dongfang DING, Conghui LI, Yi ZHU

**Affiliations:** 1 The Fifth Affiliated Hospital of Zhengzhou University; 2 Academy of Medical Sciences, Zhengzhou University, Zhengzhou, Henan; 3 Department of Rehabilitation Medicine, Hainan Cancer Hospital, Haikou, Hainan; 4 School of Rehabilitation Sciences and Engineering, University of Health and Rehabilitation Sciences, Qingdao, Shandong, China

**Keywords:** activities of daily living, cognitive function, cross-sectional study, diabetes, elderly

## Abstract

**Objectives:**

To investigate the association between diabetes and cognitive dysfunction in the elderly population, and examine the impact of cognitive dysfunction on level of activities of daily living (ADL) in patients with diabetes.

**Methods:**

Data analysis was conducted on 2,951 individuals aged over 65 years from the Chinese Longitudinal Healthy Longevity Survey cohort. Propensity score matching was utilized to mitigate selection bias. Multivariate binary logistic regression was performed to analyse the association between diabetes and cognitive dysfunction in the study subjects. In addition, the relationship between ADL and cognitive function in patients with diabetes was analysed using the Wilcoxon rank-sum test.

**Results:**

A significant association (*p* = 0.017) was found between diabetes and the occurrence of cognitive dysfunction in older adults. Subgroup analyses revealed that diabetes patients with cognitive dysfunction exhibited a worse ADL dependence compared with those without cognitive dysfunction (*p* < 0.001).

**Conclusion:**

These findings indicate that diabetes is associated with cognitive dysfunction in older adults. Meanwhile, there is an association between cognitive impairment and ADL level in subjects with diabetes. As such, healthcare professionals should pay close attention to the occurrence of cognitive dysfunction and ADL decline during diagnosis and treatment, and proactive prevention and intervention strategies should be implemented.

LAY ABSTRACT

This study investigated the association between cognitive dysfunction and diabetes among Chinese older adults aged 65 years and over. In order to make the data more comparable, a new method was used to adjust the imbalance between the diabetic population and the non-diabetic population. The results showed that diabetes was associated with cognitive dysfunction and decreased ability in activities of daily living in older adults. Therefore, healthcare personnel should pay close attention to the occurrence of cognitive dysfunction and decline in activities of daily living in the process of diagnosis and treatment, and put in place active prevention and intervention strategies.

Over the past 20 years, China has undergone a significant demographic transition, evolving into an ageing society with approximately 264 million people aged 60 years and above (1). This demographic shift necessitates increased research attention to address the well-being of the elderly population. Independent physical and cognitive abilities are acknowledged as crucial components contributing to healthy life expectancy in older adults (2).

Diabetes is a chronic disease that occurs either when the pancreas does not produce enough insulin or when the body cannot effectively use the insulin it produces (3). Insulin is a hormone that regulates blood glucose. Hyperglycaemia is a common effect of uncontrolled diabetes and, over time, leads to serious damage to many of the body’s systems, especially the nerves and blood vessels. China has the highest number of elderly diabetic patients globally, and projections indicate that, by 2045, the number of older adults with type 2 diabetes will exceed 78 million (4). Diabetes and its complications collectively influence multiple dimensions of health-related quality of life (5), including cognition and activities of daily living (ADL).

Cognitive dysfunction emerges as a prevailing neuropsychiatric disorder among elderly people, imposing significant social and economic burdens (6). Epidemiological studies have demonstrated that patients with diabetes are more susceptible to cognitive dysfunction compared with the general middle-aged and elderly populations (7, 8). A recent Chinese cross-sectional study revealed a stronger association between diabetes and cognitive impairment in people aged 45–59 years, while it lacked significance in individuals over 60 years old (9). In addition, patients with diabetes have substantial impairment in physical function (10, 11). Functional capacity is usually measured by ADL, which includes basic self-care abilities, such as bathing, dressing, feeding, and indoor activities (12). Measurement of ADL is important, because they are predictors of admission to nursing homes, hospitalization, and use of paid homecare (13). A recent study from Mexico found an association between diabetic cognitive dysfunction and living activities ability (14). However, further robust evidence is required to confirm this association (15).

This study, based on an extensive database, the Chinese Longitudinal Healthy Longevity Survey (CLHLS), aims to investigate the relationship between diabetes and cognitive dysfunction in older adults, aged 65 years and above, in China. Furthermore, the study explores the relationship between cognitive function and ADL in the diabetic population. The findings aim to enhance clinicians’ understanding of functional dysfunction in patients with diabetes, ultimately facilitating the design of targeted treatment programmes to improve cognitive and functional status.

## METHODS

### Study population

The study population was drawn from the CLHLS, a nationwide survey encompassing 23 provinces, municipalities and autonomous regions in China. The respondents included in the survey were aged 65 years and above. The questionnaire included basic information about the older adults and their families, socioeconomic background and family structure, self-assessment of health and quality of life, cognitive function, psychological characteristics, daily activities, etc. (2). The CLHLS study obtained ethical approval from the Biomedical Ethics Committee of Peking University (IRB00001052-13074). Detailed information about the CLHLS can be accessed at: https://opendata.pku.edu.cn/dataverse/CHADS.

The current study involved a secondary analysis of data collected in 2018. From a total of 3463 participants, 2951 individuals with complete data on diabetes and cognitive function were selected. To define diabetes, the criteria of the World Health Organization (WHO) and the American Diabetes Association (ADA) were applied, based on the measurement of blood glucose levels (16). Participants aged 65 years and above were considered for inclusion in the study, and complete data on sex, body mass index (BMI), ADL, and other covariates listed in Table SI were required. The study adhered to the Enhanced Reporting of Observational Studies in Epidemiology (STROBE) guidelines for reporting.

### Measurement of cognitive function

Cognitive function was assessed through home interviews, utilizing the Chinese version of the Mini-Mental State Examination (MMSE) questionnaire. The validity and reliability of this questionnaire have been validated in previous studies (17, 18). Cognitive dysfunction in this study was defined using the criteria of Zhang et al. (17) and Chen et al. (19), whereby a MMSE score < 18 indicated cognitive dysfunction. Additional information regarding the MMSE scores of the participants is shown in Table SII.

### Measurement of activities of daily living

ADL were evaluated based on the total scores for tasks such as bathing, dressing, using the bathroom, indoor transfer, continence, and feeding. Each item’s assessment categorized respondents into “no need for help at all” (1 point), “need for help from one person” (2 points), or “need for help from 2 or more people” (3 points). Higher scores on the ADL scale indicated higher functional dependency and greater need for external care or support from family members or caregivers. The ADL scale demonstrated good internal consistency (Cronbach’s 0.818) (2) in the 2018 CLHLS sample.

### Covariates

According to the previous studies (20, 21), relevant covariates were included in the analysis to account for potential biases: (*i*) Demographics, including sex, type of residence (urban and rural), age, years of education and body mass index (BMI); (*ii*) Psychological factors, represented by psychological well-being (PWB) (22), which is the total score of 7 questions ranging from 7 to 35, including being optimistic about things, keeping things neat, feeling afraid or anxious, feeling lonely, making your own decisions, feeling useless as you get older, and feeling happier when you were younger, with higher scores indicating better well-being; (*iii*) health-related behaviours, including smoking, drinking and exercising. Participants were considered “yes” if they had or were still engaged in these behaviours; (*iv*) community support (23), calculated by the total score of 9 services (personal care, home visits, psychological counselling, daily shopping services, social entertainment, legal assistance, medical education, neighbourhood relations, and other services) provided by the community, with a score of 1 for those who have this support and 0 for those who do not; (*v*) disease that may affect cognition in older adults (24), including hypertension, cardiopathy, cerebrovascular disease, pulmonary disease, dysaudia and somnipathy. ADL was also considered as a covariate.

### Statistical analysis

Propensity score matching (PSM) (25) was utilized to mitigate selection bias in observational clinical studies. PSM transformed multidimensional covariates into a single propensity score to match 2 groups of subjects with similar propensity scores, achieving balanced and comparable covariate distribution between the groups. The propensity score (PS) for diabetic vs non-diabetic participants were calculated using the logit model, and matching was performed at a ratio of 1:8 using the greedy nearest neighbour algorithm with a calliper width of 0.02. This resulted in a diabetic group and matched non-diabetic group. The PS for grouping was based on the sum of all covariates, so the occurrence of a single covariate *p* < 0.05 after PSM was allowed (25). Multivariate binary regression analysis was employed to examine the association between diabetes and cognitive dysfunction.

Subgroup analysis involved stratifying the diabetic population based on MMSE scores to evaluate the relationship between cognitive dysfunction and ADL in patients with diabetes. The Wilcoxon rank-sum test was used to examine the association between cognitive dysfunction and ADL in individuals with diabetes.

The Kolmogorov–Smirnov (KS) test checked the normal distribution of continuous variables, and appropriate statistical tests (corrected *t*-test or Wilcoxon rank-sum test) applied accordingly. The χ^2^ test was used for categorical variables. A 2-tailed *p-*value < 0.05 was considered statistically significant. The absolute standardized mean differences (ASMD) were used to evaluate the balance between groups, with an ASMD < 0.1 indicating well-balanced covariates (26). RStudio (version 4.3.1; RStudio, Boston, MA, USA) was utilized for PSM and data analysis.

## RESULTS

### Demographic data of the studied subjects

A total of 2951 participants were included in the study, with a diabetes group (*n* = 293) and a matched non-diabetes group (*n* = 1,635) obtained through 1:8 PSM. The mean age of the subjects was 80 years, with males accounting for 50.5%. The majority of participants resided in rural areas (88.2%). The proportion of men in the diabetic group (43.6%) was slightly lower than that in the non-diabetic group (51.2%). Detailed demographic information is shown in Table SIII.

### Propensity score matching effect between subjects with or without diabetes

**Fig. 1:**
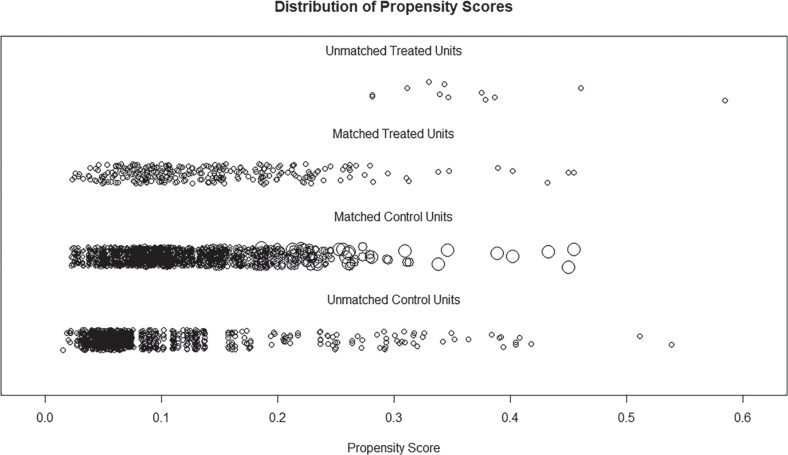
Distribution of propensity scores. The sample is represented by *circles*, while the abscissa denotes the propensity score. The intermediate 2 rows display samples from the matched intervention and control groups, whereas the top and bottom rows showcase unmatched samples.

**Fig. 2:**
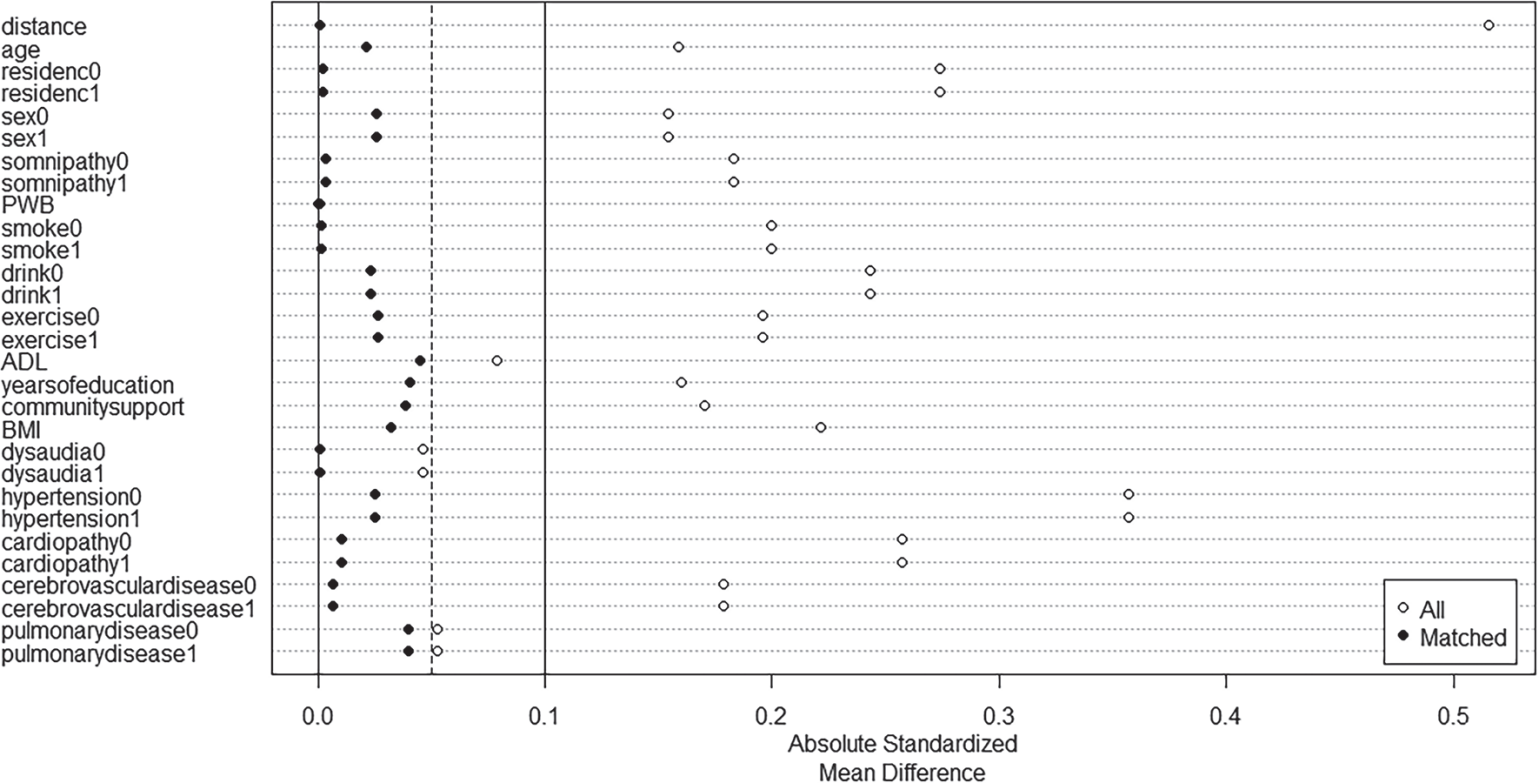
Absolute standardized mean difference. The small dots in the figure represent the ASMD before and after PSM for each covariate. Hollow dots represent ASMD before PSM and black dots represent ASMD after PSM. Before PSM, most of the hollow dots were larger than 0.1, while after PSM, all of the black dots were smaller than 0.1.

A non-parsimonious multivariable logistic regression model was employed to calculate the PS based on diabetes as the independent variable and 17 baseline variables as covariates. Fig. 1 displays the distribution of PS values before and after matching. The propensity score distributions of the diabetic and non-diabetic groups after matching were highly overlapping, indicating that the matching quality of the PSM model was acceptable. Fig. 2 illustrates the ASMD values before and after PSM, which were less than 0.1 for each factor after PSM, demonstrating improved balance between the groups after matching. Additional ASMD scores before and after PSM are shown in Table SI.

### Baseline characteristics after propensity score matching

PSM analysis successfully matched baseline characteristics between the diabetes and non-diabetes groups (Table SI). Before matching, the diabetes group’s age (80 vs 78 years, *p* = 0.007) was significantly lower than that of the non- diabetes group, and sex differences were also statistically significant (*p* = 0.011). However, after PSM, these differences in age (*p* = 0.109) and sex (*p* = 0.181) were substantially reduced. Before PSM, significant differences (*p* < 0.001) were observed in somnipathy between the diabetes and non-diabetes groups, which were mitigated after PSM (*p* = 0.112). Other variables, including BMI, smoking, alcohol consumption, exercise, years of education, community support, and diabetes comorbidity survey indicators, showed significant differences between the groups before PSM, but became comparable after matching. Before PSM, the ASMD values of each baseline characteristic were greater than 0.1 except PWB, ADL, dysaudia and pulmonary disease. The ASMD values after PSM were all less than 0.1, indicating that group comparisons were comparable and baseline characteristics were balanced between the diabetic and non-diabetic groups. The specific *p*-values and ASMD for each baseline characteristic are shown in Table SI and Fig. 2.

### Association between other related factors and cognitive dysfunction after propensity score matching

To explore factors associated with cognitive dysfunction in older adults, a multivariate binary regression analysis was performed on the subjects after PSM (Table SIV). The analysis was adjusted for several factors, including age, type of residence, sex, PWB, smoking, alcohol consumption, exercise, ADL dependence, community support, BMI, somnipathy, dysaudia, hypertension, diabetes, cerebrovascular diseases, cardiopathy, and pulmonary disease. After controlling for the differences within groups using PSM, the results indicated that patients with diabetes were more likely to have cognitive dysfunction than those without diabetes (*p* = 0.017). In addition, age (*p* < 0.001), PWB (*p* < 0.001), years of education (*p* = 0.046), ADL dependence (*p* < 0.001) and dysaudia (*p* < 0.001) were associated with cognitive dysfunction, irrespective of diabetes status.

### Decreased activity of daily living level in diabetic patients with cognitive dysfunction

Subgroup analysis was performed to investigate the association between cognitive dysfunction and ADL dependence in patients with diabetes, both in the original data and after PSM. In the original data, diabetic patients were divided into a non-cognitive dysfunction group (*n* = 259) and a cognitive dysfunction group (*n* = 46) based on cognitive dysfunction status. The Wilcoxon rank-sum test demonstrated a significant difference (*p* < 0.001) between the 2 groups, indicating that cognitive dysfunction in diabetic patients correlated with ADL dependence. After PSM, the data showed consistent results, with a significant association (*p* < 0.001) between cognitive dysfunction and ADL dependence in diabetic patients.

## DISCUSSION

The current study aimed to investigate the association between cognitive dysfunction and diabetes in a large sample of Chinese older adults, and to explore the impact of cognitive impairment on ADL in diabetic patients. The results demonstrated a significant association between diabetes and cognitive dysfunction in elderly individuals, with diabetic patients experiencing decreased ADL levels. These findings underscore the impact of diabetes on both functional and cognitive impairment in the elderly population.

Consistent with these findings, a recent large Canadian cohort study reported an increased risk of dementia in people newly diagnosed with diabetes (27). Several cohort studies also suggest that older patients with diabetes exhibit poor cognitive performance in various domains, including information processing speed, memory, attention, and executive function compared with non-diabetic controls (28, 29). These studies provide support for our observation that diabetes is associated with cognitive dysfunction in elderly patients. Numerous proposed mechanisms have been suggested to explain cognitive dysfunction in diabetes, including cerebral insulin resistance and amyloidosis, which are considered major contributors to cognitive dysfunction induced by diabetes (30). Insulin and insulin resistance can affect brain myelin content, which is associated with cognitive decline in diabetes (31). In addition, impaired fasting glucose induced by diabetes may lead to increased oxidative levels and reduced antioxidant defence mechanisms, resulting in functional or cellular brain defects that lead to cognitive dysfunction (32). Vascular damage caused by pathoglycaemia may also be an important factor leading to cognitive dysfunction (33).

Notably, the association between diabetes and cognitive dysfunction may vary with age. Zhang et al. (9) based on the China Health and Retirement Longitudinal Study, found significant cognitive decline in middle-aged individuals (45–59 years) with diabetes, but no significant association for individuals aged over 60 years. Conversely, Lyu et al. (34) reported a 2.1-fold higher risk of cognitive dysfunction in elderly patients with diabetes aged 75 years and older compared with those under 75 years of age. The current study focused on individuals aged 65 years and older and provided valuable insights, emphasizing the continued importance of paying attention to cognitive dysfunction in older diabetic patients. At the same time, the above contradictory results may also be related to the investigation population. Cultural, dietary and ethnic differences may all contribute to this. Therefore, large-scale cross-racial investigations deserve more attention.

Multivariate binary logistic regression analysis in the current study revealed that age, PWB, years of education, and other included confounding factors may also influence cognitive dysfunction. Age emerged as a statistically significant risk factor for cognitive dysfunction, consistent with the findings of Brewster et al. (35). Moreover, Bento-Torres et al. (36) observed significantly worse cognitive performance in less educated populations and advocated for early inclusion of formal education in preventive public health agendas. Dysaudia, a cause of cognitive decline, was identified as a risk factor for cognitive dysfunction (37). The 2017 and 2020 Lancet Commission on Dementia identified dysaudia as the single largest modifiable risk factor for dementia, estimating an 8% reduction in dementia prevalence if dysaudia were eliminated (35). In the previous analysis of this longitudinal CLHLS study, researchers found that higher PWB was associated with lower odds of developing cognitive dysfunction (38). The positive association between higher PWB and lower odds of developing cognitive dysfunction aligns with the results of the current study, indicating a complex and bidirectional relationship of PWB with physical and neuronal health (39).

In subgroup analysis, the current study found that ADL level was associated with cognitive dysfunction. Decreased ADL level not only increases the incidence of cognitive dysfunction, but also cognitive decline significantly impacts ADL and quality of life, in elderly patients (40). A meta-analysis showed that cognitive training interventions had a positive effect on ADL level in people with cognitive dysfunction, reinforcing the importance of considering both cognitive dysfunction and ADL in the management of diabetes (41). Therefore, in the clinical diagnosis and treatment of diabetes, in addition to cognitive dysfunction, ADL also deserves attention.

The increasing attention to cognitive dysfunction in patients with diabetes emphasizes the need for evaluating cognitive function in clinical diagnosis and treatment, particularly in those over 65 years old. In addition, cognitive function in elderly patients with diabetes may be affected by various factors and related risk scores can improve attention to cognitive function and enable timely interventions for high-risk groups (42). While treatment recommendations for diabetic patients are currently primarily based on medication, patient self-management and interventions targeting cognitive dysfunction should be integral components of treatment strategies (43). Family members or primary caregivers of elderly diabetic patients should encourage their participation in daily activities to the extent possible and support them in completing activities independently or with partial assistance. Focusing on the psychological well-being of this group, increasing family time, engaging them in social activities, providing relevant education, and helping them adjust to social changes are all recommended. Regular psychological evaluations and timely psychological interventions should also be implemented.

However, the current study has some limitations. Firstly, as a cross-sectional study, it cannot confirm a causal relationship between diabetes and the increased risk of cognitive dysfunction in elderly patients. Future cohort studies are needed to clarify causality. Secondly, bias may be introduced due to differences in diabetes duration and treatment. This limitation should be considered in the design of future databases to ensure comprehensive information collection. Lastly, while 17 confounding factors were included in this study based on relevant previous evidence, there may be other unconsidered factors that could have influenced the findings.

In conclusion, this study contributes valuable epidemiological evidence supporting the association between diabetes and cognitive dysfunction in Chinese older adults. Diabetes was found to be significantly associated with cognitive dysfunction in elderly patients, along with increasing age, decreased ADL level, lower psychological well-being, lower years of education, and dysaudia. Furthermore, cognitive dysfunction was linked with decreased ADL level in individuals with diabetes. Thus, these findings underscore the importance of paying greater attention to both cognitive dysfunction and ADL in the clinical diagnosis and treatment of diabetes, especially in elderly patients.

## Supplementary Material

ASSOCIATION BETWEEN COGNITIVE DYSFUNCTION AND DIABETES IN PATIENTS OVER 65 YEARS OLD: A CROSS-SECTIONAL STUDY USING PROPENSITY SCORE MATCHING
