# Supplementary material for: ASSOCIATION BETWEEN COGNITIVE DYSFUNCTION AND DIABETES IN PATIENTS OVER 65 YEARS OLD: A CROSS-SECTIONAL STUDY USING PROPENSITY SCORE MATCHING
Source: J Rehabil Med. 2024 Feb 21;56:18372. doi: 10.2340/jrm.v56.18372 (PMC10896218; doi:10.2340/jrm.v56.18372)
Supplement: ASSOCIATION BETWEEN COGNITIVE DYSFUNCTION AND DIABETES IN PATIENTS OVER 65 YEARS OLD: A CROSS-SECTIONAL STUDY USING PROPENSITY SCORE MATCHING [file JRM-56-18372-s1.pdf]

Supplementary material has been published as submitted. It has not been copyedited, typeset or checked for scientific content by Journal of Rehabilitation Medicine

Table SI Details About the Chinese Version of MMSE.

Dimensions	Item	Scores
Orientation	1.what time of day is it right now? morning, noon, afternoon, or evening?	1
	2.what is the animal year of this year?	1
	3.what is the date of the mid-autumn festival?	1
	4.what is the season right now？ spring, summer, fall, or winter?	1
	5.what is the name of this county or district?	1
	6.Please tell me as many foods as a man can eat. (Take a minute) (1 point for each food and 7 points for those who name 7 or more foods).	7
Registration	7–9. Please repeat these three objects: table, apple, clothes.	3
Attention and calculation	10–14. If you have $20 and you spent $3, how many dollars do you have left? You then have to spend another $3 from the money you have left, and continue to spend $3 until you are asked to stop.	5
	15.Please draw the figure on B Card.	1
Recall	16–18. Please name the three things I just asked you to repeat. (table, apple, clothes).	3
Language	19–20. Please name pen and watch.	2
	21. Please repeat what I said below: "As you sow, so you reap."	1
	22–24. I will give you a piece of paper. Please take this paper in your right hand, fold it in half with both hands, and put it on the floor.	3

Table SII Clinical Characteristics of Subjects

Clinical variables	Total (N=2951)	Non-diabetes(N=2646)	diabetes (N=305)	P value
Age M (P25, P75)	80(74, 86)	80(74, 86)	78(74, 84)	0.007
PWB M (P25, P75)	26(24, 28)	26(24, 28)	26(23, 28)	0.987
ADL M (P25, P75)	6(6, 6)	6(6, 6)	6(6, 6)	0.118
BMI M (P25, P75)	22.1(19.6, 24.8)	22.0(19.5, 24.7)	22.9(20.5, 26.0)	<0.001
Years of education M (P25, P75)	0(0, 0)	0(0, 0)	0(0, 2)	0.003
Residence n (%)				<0.001
Rural 0	2603(88.2)	2365(89.4)	238(78.0)	
Urban 1	3489(11.8)	281(10.6)	67(22.0)	
Male n (%)	1489(50.5)	1356(51.2)	133(43.6)	0.011
Smoke n (%)	507(17.2)	472(17.8)	35(11.5)	0.005
Drink n (%)	482(16.3)	452(17.1)	30(9.8)	0.001
Exercise n (%)	889(30.1)	771(29.1)	118(38.7)	0.001
Community support M (P25, P75)	1(0, 3)	1(0, 2)	2(0, 3)	<0.001
Somnipathy n (%)	405(13.7)	343(13.0)	62(20.3)	<0.001
Dysaudia n (%)	1034(35)	921(34.8)	113(37.0)	0.437
Hypertension n (%)	1126(38.2)	961(36.3)	165(54.1)	<0.001
Cerebrovascular n(%)	292(9.9)	244(9.2)	48(15.7)	<0.001
Cardiopathy n (%)	416(14.1)	343(13.0)	73(23.9)	<0.001
pulmonary disease n(%)	331(11.2)	292(11.0)	39(12.8)	0.359

Notes: *A significant difference between groups. Categorical values are shown as n (%). Continuous variables presented skewed data and were expressed as medians and interquartile ranges.

Abbreviations: PWB, psychological well-being; ADL, activities of daily living; BMI, body mass index;

Table SIII Baseline Characteristics Before and After Propensity Score (PS) Matching

	Before PSM				After PSM			
	Non-diabetes (N=2646)	diabetes (N=305)	P value	ASMD	Non-diabetes (N=1635)	Diabetes (N=293)	P value	ASMD
Age M (P25, P75)	80(74, 86)	78(74, 84)	0.007	0.1588	79(74, 86)	78(74, 85)	0.109	0.0216
PWB M (P25, P75)	26(24, 28)	26(23, 28)	0.987	<0.001	26(24, 28)	26(23, 28)	0.876	<0.001
ADL M (P25, P75)	6(6, 6)	6(6, 6)	0.118	0.0792	6(6, 6)	6(6, 6)	0.015	0.0449
BMI M (P25, P75)	22.0(19.5, 24.7)	22.9(20.5, 26.0)	<0.001	0.2218	22.3(19.9, 25.0)	22.8(20.5, 26.0)	0.440	0.0320
Years of education M (P25, P75)	0(0, 0)	0(0, 2)	0.003	0.1599	0(0, 0)	0(0, 2)	0.454	0.0404
Residence n (%)			<0.001				0.002*	
Rural 0	2365(89.4)	238(78.0)		0.2741	1432(87.6)	236(80.5)		0.0025
Urban 1	281(10.6)	67(22.0)		0.2741	203(12.4)	57(19.5)		0.0025
Male n (%)	1356(51.2)	133(43.6)	0.011	0.1541	798(48.8)	130(44.4)	0.181	0.0260
Smoke n (%)	472(17.8)	35(11.5)	0.005	0.1996	244(14.9)	35(11.9)	0.213	0.0016
Drink n (%)	452(17.1)	30(9.8)	0.001	0.2433	229(14.0)	30(10.2)	0.099	0.0234
Exercise n (%)	771(29.1)	118(38.7)	0.001	0.1961	527(32.2)	110(37.5)	0.087	0.0267
Community support M (P25, P75)	1(0, 2)	2(0, 3)	<0.001	0.1702	1(0, 3)	1(0, 3)	0.368	0.0389
Somnipathy n (%)	343(13.0)	62(20.3)	<0.001	0.1830	244(14.9)	55(18.8)	0.112	0.0034
Dysaudia n (%)	921(34.8)	113(37.0)	0.437	0.0464	590(36.1)	110(37.5)	0.681	0.0010
Hypertension n (%)	961(36.3)	165(54.1)	<0.001	0.3568	707(43.2)	153(52.2)	0.005*	0.0250
Cerebrovascular n(%)	244(9.2)	48(15.7)	<0.001	0.1789	165(10.1)	42(14.3)	0.040*	0.0064
Cardiopathy n (%)	343(13.0)	73(23.9)	<0.001	0.2571	236(14.4)	64(21.8)	0.002*	0.0104
pulmonary disease n(%)	292(11.0)	39(12.8)	0.359	0.0524	178(10.9)	38(13.0)	0.347	0.0397

Notes: *A significant difference between groups. Categorical values are shown as n (%). Continuous variables presented skewed data and were expressed as medians and interquartile ranges.

Abbreviations: PWB, psychological well-being; ADL, activities of daily living; BMI, body mass index; ASMD, Absolute Standardized Mean Difference.

Table SIV Multivariate Logistic Regression Analysis in Patients Based on the PSM

Variables	Non- Cognitive dysfunction (N=1692)	Cognitive dysfunction (N=236)	OR	95% CI	p-Value
Age M (P25, P75)	78.00(74.00, 85.00)	88.50(83.00, 94.75)	1.083	(1.061, 1.106)	<0.001*
Residence n (%)			0.688	(0.385, 1.229)	0.206
Rarul 0	1454(85.9)	214(90.7)			
Urban 1	238(14.1)	22(9.3)			
Male n (%)	853(50.4)	75(31.8)	0.849	(0.577, 1.248)	0.405
PWB M (P25, P75)	26(24, 28)	22(21, 25)	0.814	(0.767, 0.863)	<0.001*
Years of education M (P25, P75)	0(0, 0)	0(0, 0)	0.918	(0.844, 0.998)	0.046*
Smoke n (%)	260(15.4)	19(8.1)	0.852	(0.461, 1.575)	0.610
Drink n (%)	248(14.7)	11(4.7)	0.528	(0.254, 1.095)	0.086
Exercise n (%)	599(35.4)	38(16.1)	0.781	(0.505, 1.205)	0.264
ADL M (P25, P75)	6(6, 6)	7(6, 10)	1.302	(1.205, 1.406)	<0.001*
Community support M (P25, P75)	1(0, 3)	1(0, 2)	0.957	(0.880, 1.040)	0.304
Somnipathy n (%)	263(15.5)	36(15.3)	0.752	(0.470, 1.203)	0.235
BMI M (P25, P75)	22.5(202, 25.3)	20.8(18.7, 23.9)	0.994	(0.954, 1.034)	0.752
Dysaudia n (%)	513(30.3)	187(79.2)	4.073	(2.802, 5.919)	<0.001*
Hypertension n (%)	764(45.2)	96(40.7)	0.951	(0.666, 1.358)	0.781
Diabetes n (%)	249(14.7)	44(18.6)	1.727	(1.103, 2.705)	0.017*
Cardiopathy n (%)	276(16.2)	26(11.0)	0.695	(0.412, 1.174)	0.174

Cerebrovascular n (%)	173(10.2)	34(14.4)	1.573	(0.931, 2.657)	0.091
pulmonary disease n (%)	183(10.8)	33(14.0)	1.379	(0.832, 2.286)	0.212

Notes: *A significant difference between groups. Categorical values are shown as n (%). Continuous variables presented skewed data and were expressed as medians and interquartile ranges.

Abbreviations: PWB, psychological well-being; ADL, activities of daily living; BMI, body mass index.
